# Ubiquitous impact of natural selection on nucleotide diversity in 178 species of primates

**DOI:** 10.1186/s13059-026-04093-z

**Published:** 2026-05-01

**Authors:** Bjarke Meyer Pedersen, Juraj Bergman, Vasili Pankratov, Mikkel Heide Schierup

**Affiliations:** Bioinformatics Research Centre, Department of Molecular Biology and Genetics, Aarhus University, Aarhus C., DK 8000 Denmark

## Abstract

**Background:**

Genetic diversity shows great variation across the genome. To investigate the major evolutionary forces causing this variation in primates, we analyse genomic diversity in 178 species in coordinates of the human genome reference to enable direct comparisons.

**Results:**

Across species, we find that genetic diversity decreases with declining recombination rates, with an average difference of 40% between high and low recombination regions (ranging from 0.76% to 79%). The magnitude of this effect scales with the estimated effective population size (Ne), being more pronounced in species with larger Ne. Using forward-in-time simulations, we show that these patterns are consistent with the expectations under both background selection and genetic hitchhiking and cannot arise under neutrality. We observe a skew in the site frequency spectrum toward rare variants in low-recombination regions in all species where this could be assessed, which our simulations show is consistent with both forms of linked selection.

**Conclusions:**

Understanding the importance of linked selection across the genome and between species has important implications for how genetic variation is shaped and the assumptions we use to infer population structure, demography, and selection. Our results show that linked selection is pervasive across primate genomes and leaves very few regions to evolve neutrally, even in regions of high recombination.

**Supplementary Information:**

The online version contains supplementary material available at 10.1186/s13059-026-04093-z.

## Background

Nucleotide diversity varies greatly across the genomes of primates [1, 2] and other species [3, 4], and these patterns of diversity often differ between species. The variation in diversity across the genome is due to the interplay of multiple evolutionary forces, including mutation rate, natural selection, population demography, and recombination [5, 6]. While mutation rate heterogeneity affects nucleotide diversity patterns, especially at small scales and for sex chromosomes [7], studies of germline mutations show a relatively uniform distribution of new mutations along the genome [8] and a limited range of mutation rates between species [9]. On the other hand, the long-term effect of natural selection on nucleotide diversity is modulated by the effective population size and recombination rate of a species, making it challenging to assess its importance in shaping genome-scale diversity patterns.

When natural selection acts on a variant, either positively selecting for beneficial alleles or negatively selecting against deleterious alleles, diversity in adjacent genomic regions is also affected due to shared genealogical history. This effect, termed linked selection, can be divided into background selection (BGS), which is the removal of neutral diversity linked to deleterious variants [10], and genetic hitchhiking, which is the removal of neutral diversity linked to positively selected alleles [11, 12]. Higher recombination rates allow neutral variants to escape the effects of nearby selection, leading to a positive correlation between recombination rate and nucleotide diversity [6, 13–17]. Importantly, the magnitude of this relationship reflects both the prevalence and strength of selection affecting linked neutral diversity [18, 19].

Most novel mutations are expected to be deleterious, and thus, background selection likely affects nucleotide diversity across much of the genome [20]. However, while it occurs relatively frequently, each instance of background selection has modest effects on linked neutral diversity. In contrast, positive selection occurs more rarely but can have dramatic consequences when it does, causing sharp reductions of nearby diversity [11].

The impacts of BGS and genetic hitchhiking on nucleotide diversity vary with population size in fundamentally different ways. For BGS, theory predicts that selection against deleterious mutations most strongly affects linked neutral diversity when the selection coefficient is approximately s ≈ 1/Ne [10, 21]. In contrast, positive selection tends to have an increasingly stronger impact on linked diversity as population size increases through multiple mechanisms. When a beneficial mutation sweeps to fixation in a larger population, it affects a greater absolute amount of linked neutral variation. First, because larger populations inherently maintain higher baseline genetic diversity [12]. Second, in larger populations, weakly beneficial mutations are more likely to overcome stochastic loss and reach frequencies where selection predominates over drift [20, 22]. Finally, larger populations experience higher total mutation rates per generation, reducing the expected waiting time for adaptive mutations to arise [11, 12]. These population size-dependent effects might create distinct genomic signatures of hitchhiking versus background selection. Using a multi-species dataset allows for investigating how the total amount of linked selection changes between species and scales with the effective population size.

Here, we performed a comparative study of nucleotide diversity, recombination rate, and effective population size using genome-wide diversity data from 178 primate species. The phylogenetic span and genomic synteny of the studied species allowed us to provide an unbiased view of the relationship between linked selection and nucleotide diversity. We found that the observed patterns in primates are best explained by pervasive linked selection, with the magnitude of linked selection scaling with effective population size. We then used forward-in-time simulations to establish that these empirical patterns are consistent with theoretical expectations under linked selection, but not under neutral evolution.

## Results

### The consistent relationship between nucleotide diversity and recombination across the entire primate phylogeny

We extracted 3.8 million 100 kb windows with homology to the human reference genome from nucleotide diversity data from 178 primate species. On average, the lift-over of each primate reference sequence covered ~ 71% of the human reference, with a median number of 23,547 100 kb windows lifted per species, containing an average of 4.7 million variants per species. Our filtering procedure retained 54–96% of windows across species, with retention generally decreasing with phylogenetic distance from humans. This raises the concern that windows excluded due to poor lift-over might be systematically different from retained windows. Specifically, excluded windows might represent faster-evolving regions with higher diversity that also tend to have higher recombination rates. To address this, we examined the relationship between retention and recombination rate and found a slight positive Spearman’s rank correlation [Great apes: 0.0454, New World monkeys: 0.194, Old World monkeys: 0.1574, Prosimians: 0.129] between callability and recombination rate. Therefore, regions with higher recombination rates tend to be weakly associated with liftability to human coordinates, potentially reflecting their higher gene density.

**Fig. 1:**
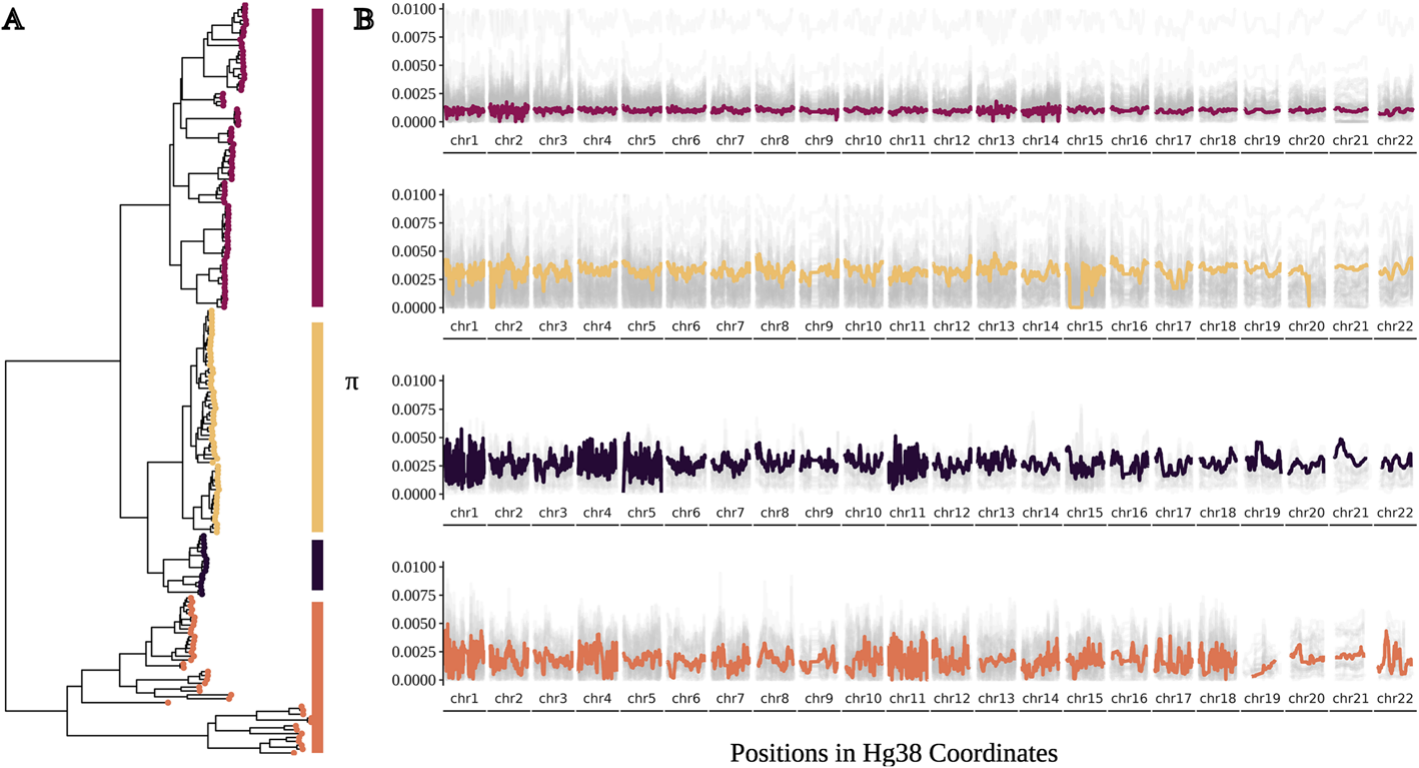
**A** A phylogenetic tree of the species, clustered into 4 groups: New World monkeys (purple), Old World monkeys (yellow), Apes (black) and Prosimians (Orange). **B** Average nucleotide diversity within species as a rolling mean of 5 Mb windows along each chromosome in hg38 positions. Only one species per phylogenetic group is coloured (Purple: Pithecia albicans, Yellow: Macaca fascicularis, Black: Pongo abelii, Orange: Lemur catta), the rest is coloured gray in the background

The landscape of nucleotide diversity of 100 kb windows in Hg38 coordinates varied across chromosomes, species and phylogenetic groups (Fig. 1). Across primate species, nucleotide diversity, measured as the average nucleotide diversity per site (π), ranged 67-fold, from 1.30 × 10^–4^ in Alouatta palliata to 8.82 × 10^–3^ in Chiropotes albinasus. We observed higher nucleotide diversity near telomeres and a decrease towards the centromere, a phenomenon also observed in other mammalian groups [23, 24]. This pattern also mirrored the human recombination landscape with higher rates towards the telomeric regions [25–27].

We used the human pedigree-based recombination map [28], averaged over 100 kb windows, as a proxy for the regional recombination rate across all the primates (see Methods and materials). As this map is pedigree-based we avoid systematic biases in rate estimation from diversity and marker density that linkage disequilibrium-based methods might have. At finer scales spatial resolution might still be affected by SNP density when using pedigree-based methods. At a fine scale (1–10 kb), recombination rates differ even between closely related primates due to changes in the recognition sequence of the recombination-directing PRDM9 protein [29, 30]. However, when comparing the human pedigree-based recombination map [28] to the LD-based recombination map of Macaca mulatta [31], we find that at a 100 kb level the recombination rates are well conserved with a genome-wide Spearman rank correlation of 0.43 (ranging between 0.35 on chr7 to 0.58 on chr17, with a chromosome size weighted mean of 0.42) (see Additional file 2: Table S1).

**Fig. 2:**
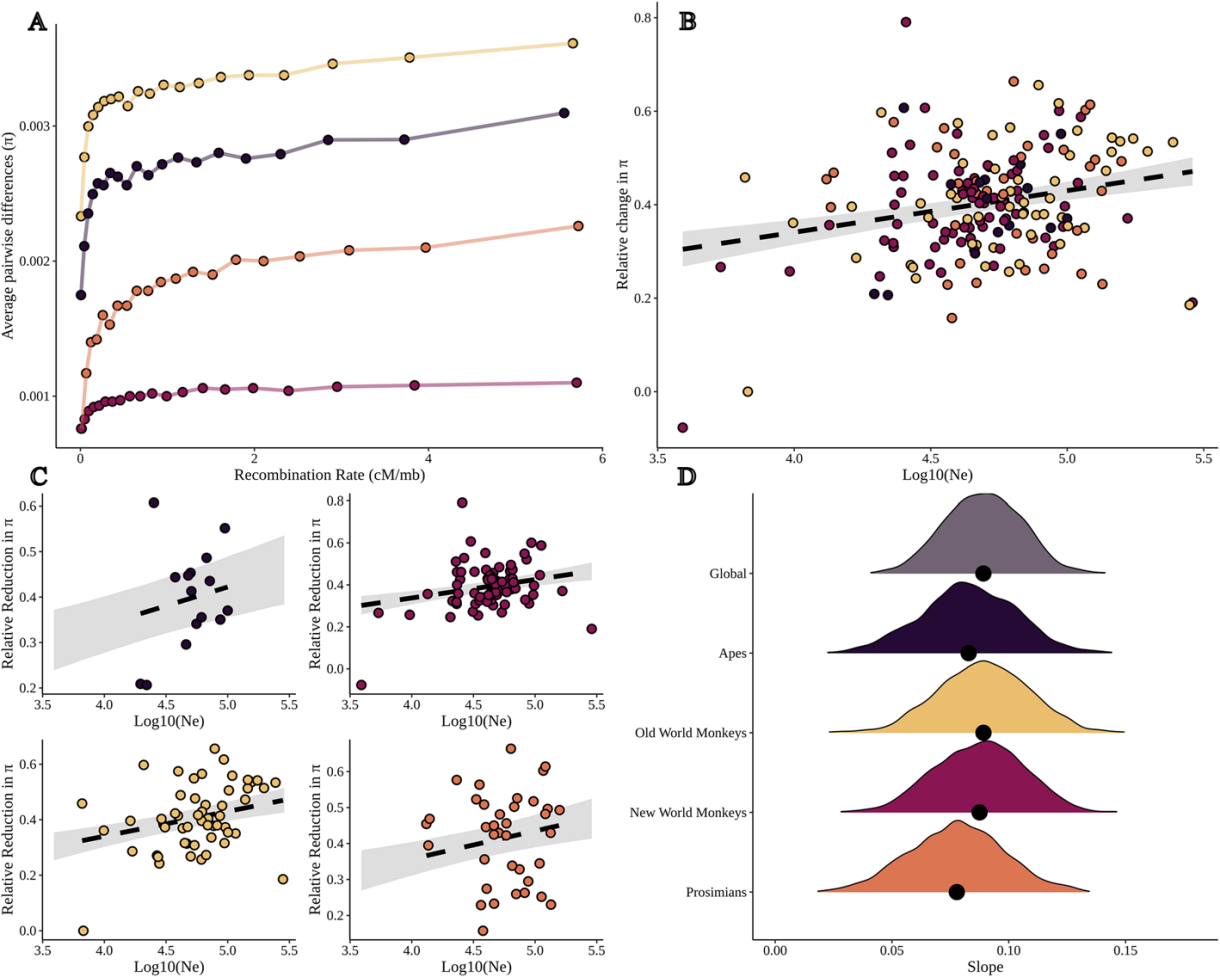
**A** The relationship between recombination rate (summarized into 20 bins of increasing rate magnitude) and average nucleotide diversity calculated across 100 kb genomic windows in four example species (Purple: Pithecia albicans, Yellow: Macaca fascicularis, Black: Pongo abelii, Orange: Lemur catta). **B** The relative change in diversity between the highest recombining bin and lowest recombining bin as a function of the estimated effective population size. Colours correspond to the phylogenetic group of each species: New World monkeys (purple), Old World monkeys (yellow), Apes (black) and Prosimians (Orange). **C** The relative change in diversity divided into 4 phylogenetic groups to show consistency across primate lineages. **D** The posterior distribution of the slopes for the relationship between the relative diversity change and effective population size

Next, we used the human recombination map to stratify each species’ genome into 20 equally sized bins of increasing recombination rate magnitude. Specifically, we ranked all the 100 kb windows by their recombination rate and divided them into 20 quantiles. We plotted the median nucleotide diversity trend across the bins for each species (Fig. 2A and Additional file 1: Figs. S1-S4). Across all species, diversity increases with recombination, but the shapes of the relationships differ. We observe a levelling out of nucleotide diversity at high recombination rates (Fig. 2A). This plateau represents a saturation effect, where neutral sites in high-recombination regions have become sufficiently decoupled from sites under selection that further increases in recombination have diminishing impacts on diversity. The recombination rate at which this levelling out is observed and the asymptotic diversity reached vary among species. Species with large effective population sizes tend to show less levelling out, as selection is likely to affect larger genomic regions in these species, leaving few to no regions unaffected by linked selection.

### The change in nucleotide diversity between high and low recombining regions increases with population size

An increase in diversity with increasing recombination is expected under both genetic hitchhiking and background selection, as both models predict a positive relationship between diversity and recombination rate, whereas neutral models do not. This expectation is confirmed by our forward-in-time simulations (Additional file 1: Figs. S1-S4). However, this effect is of unknown strength in natural populations. To address this, we estimate long-term effective population sizes for each species utilizing the established relationship between mutation rates and primate generation times [9] (Additional file 1: Text). The estimated population sizes varied across a 73-fold range (with a minimum Ne of 3,904 in Alouatta palliata and a maximum Ne of 287,675 estimated for Chiropotes albinasus), i.e., a ~ 1.15 × larger range than the range in diversity. The relative change in nucleotide diversity, estimated as the difference in median diversity between the highest and lowest recombination bin, varied 100-fold across species (Fig. 2A and Additional file 1: Figs. S1-S4). Generally, regions with low recombination rates showed, on average, 40% lower nucleotide diversity compared to highly recombining regions. Only one species, Alouatta palliata, showed a reverse trend, with lowly recombining regions having 0.76% higher diversity than highly recombining regions. In contrast, Aotus trivirgatus exhibited the most pronounced effect, with lowly recombining regions having 79% lower diversity than highly recombining regions. We used a Bayesian framework to model the relationship between the long-term population size and the relative change in diversity (Fig. 2B). We found a significantly positive relationship between the effective population size and relative change in diversity, with the 95% highest posterior density interval (HPDI) of the slope estimated to be [0.058—0.120, median = 0.089]. On average, with a twofold change in N_e_, the relative change increases by 2.68%. This empirical result is consistent with the expectations from our forward-in-time simulations, which likewise show increasing slopes of the diversity-recombination relationship with increasing Ne under both background selection and hitchhiking scenarios (Additional file 1: Figs. S6-S8). To investigate if the slope of this relationship was affected by phylogenetic distance to the human reference, we repeated this analysis for four separate phylogenetic groups of primates (Platyrrhini; New World monkeys, Cercopithecidae; Old World monkeys, Hominoidea; Apes, Strepsirrhini; Prosimians). We found no significant changes in slopes when considering different groups of primates, with HPDIs of the slope overlapping for all groups [Apes: 0.042–0.12, New World monkeys: 0.05–0.12, Old World monkeys: 0.05–0.12, Prosimians: 0.04–0.118] (Fig. 2C, D). The consistency of results across groups that differ substantially in their divergence time from humans (6 MYA for apes to 75 MYA for prosimians) provides evidence that our findings are not artifacts of using a single recombination map.

### Hierarchical modelling of the diversity-recombination relationship

**Fig. 3:**
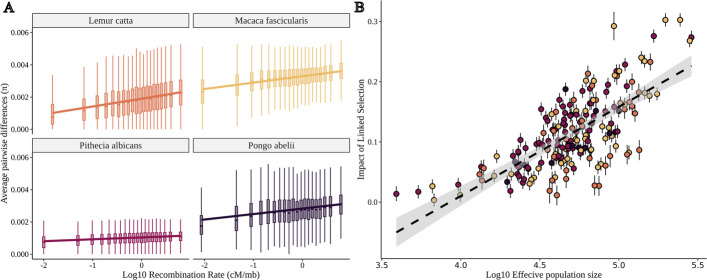
**A** Four example species (Lemur catta, Macaca fascicularis, Pithecia albicans, Pongo abelii), showing the relationship between the log-scaled recombination rate and average nucleotide diversity, summarized into 20 bins, although we used all windows in the modelling. **B** The relationship between effective population size and the rate of change from the recombination rate diversity relationship (slope)

To better understand the relationship between nucleotide diversity and recombination across the full range of recombination rates, we built a Bayesian hierarchical model that allows the slopes and intercepts of the recombination-diversity relationship to vary between species, while accounting for species-specific effective population size. Specifically, we modelled the recombination-diversity relationship using log_10_-transformed recombination rates, which yielded a linear relationship (Fig. 3A). Our Bayesian model incorporated the long-term effective population size as a global predictor of the strength of the species-specific slopes for the recombination-diversity relationship. ​​Using this framework, we observed a clear relationship between the species-specific slopes and Ne (Fig. 3B). Of the 178 species we analyzed, 173 exhibited positive 95% highest density posterior intervals (HDPIs) for the recombination-diversity relationship. The median slopes varied from 0.00335 to 0.303; with this consistent positive relationship across primate species our results provide strong evidence for the pervasive effects of linked selection across the phylogeny.

We found that the global predictor of effective population size is positively related to the slope of the diversity-recombination relationship (95% HPDI: 0.0389–0.0517), indicating that species with larger population sizes are also likely to experience more/stronger selection and are therefore less likely to have regions escaping the influence of linked selection.

### Evidence from the site frequency spectrum

**Fig. 4:**
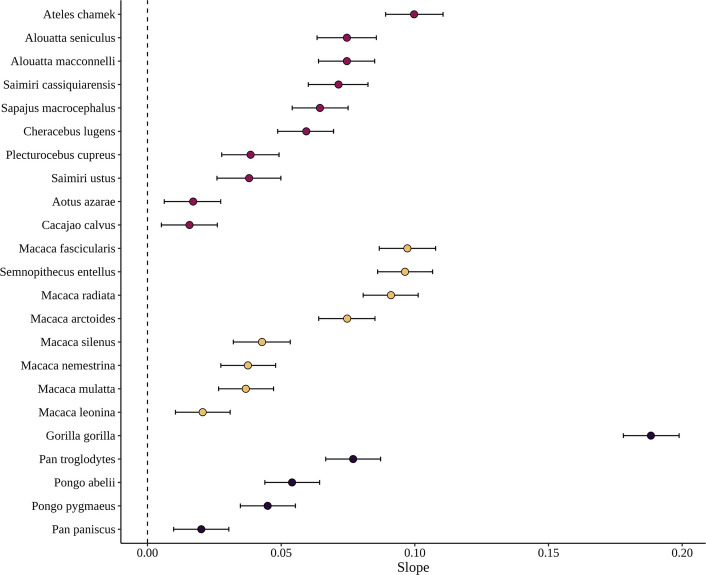
Slope of Tajima’s D ~ Recombination relationship for all species with 5 or more samples. Each species is coloured according to phylogenetic grouping

To investigate an additional line of evidence for the role of selection in shaping the relationship between nucleotide diversity and recombination, we calculated the median Tajima’s D across recombination rate bins for each species. Among species with sufficient sample sizes (n > 5), we modelled the relationship between log_10_ recombination rate and Tajima’s D. We found significantly positive slopes in all species, with medians ranging from 0.005 to 0.17, with the smallest slope found in Cacajao calvus and the largest slope found in Gorilla gorilla (Fig. 4; individual species plots in Additional file 1: Fig. S5). These positive relationships are consistent with linked selection contributing to the diversity-recombination relationship across the primate lineage. Whether the strength of the Tajima’s D-recombination relationship scales with effective population size was also investigated through simulations; however, no clear pattern was observed (Additional file 1: Fig. S13), suggesting that this statistic is not diagnostic for inferring population size effects on selection.

### Forward-in-time simulations confirm theoretical expectations

The empirical patterns we observe (a positive diversity-recombination relationship that scales with effective population size and reduced Tajima’s D in low-recombination regions) are consistent with linked selection. However, to formally establish that these patterns cannot arise under neutrality and to understand expectations under different selection regimes, we performed forward-in-time simulations using SLiM [32]. We simulated a recombining chromosome using the human recombination map [33] as implemented in the stdpopsim catalog [34] under three scenarios: neutrality, background selection, and genetic hitchhiking (see Methods and materials for details).

Under neutral evolution, our simulations produced no relationship between recombination rate and nucleotide diversity (Additional file 1: Fig. S6), as expected from theory [16]. In contrast, both background selection and hitchhiking simulations produced a consistent positive correlation between recombination rate and diversity (Additional file 1: Figs. S7-S8). The strength of this relationship increased with effective population size under both models, matching our empirical observation that species with larger Ne show stronger diversity-recombination slopes (Additional file 1: Fig. S9). The scaling factor was similar for both selection models, indicating that the diversity-recombination relationship alone cannot distinguish between background selection and hitchhiking. We also examined Tajima’s D across recombination rate bins in our simulations. Our simulations demonstrate that both background selection and genetic hitchhiking produce a positive relationship between Tajima’s D and recombination rate (Additional file 1: Figs. S11-S12). Importantly, our simulations show that neither the nucleotide diversity-recombination relationship nor the Tajima’s D-recombination relationship is produced under the neutral scenario (Additional file 1: Fig. S10).

## Discussion

### The role of mutation rate variation and recombination map divergence

While our results support linked selection as the primary driver of the observed diversity-recombination relationships, it is important to consider alternative explanations. Mutation rate heterogeneity along the genome could potentially contribute to diversity patterns [8]. However, several lines of evidence argue against this being a major confounding factor in our analysis. First, the extreme mutation rate regions identified by Jónsson et al. [8], mainly reported on the p arm of chromosome 8 and portions of chromosome 16, represent less than ~ 2% of windows in our genome-wide analysis. When using median values across ~ 30,000 100 kb windows, these hotspot regions will have minimal influence on the overall patterns. Further, recombination itself may have mild mutagenic effects due to error-prone repair of double-strand breaks. However, the effect size of recombination-associated mutagenesis (typically a 1.5- to twofold increase [28]) cannot explain the 40% average difference between high and low recombination regions that we observe, nor can it explain why this difference scales with effective population size. Furthermore, the consistency of our results across phylogenetic groups, despite their different divergence times from humans and potentially different mutation landscapes, further supports that selection, rather than mutation rate variation, drives these patterns. Additionally, while fine-scale recombination hotspots evolve rapidly due to PRDM9 sequence evolution [30], broad-scale recombination patterns show substantial conservation across evolutionary time, although the level of conservation is likely to depend on phylogenetic distance. Importantly, several factors suggest that using a single recombination map does not compromise our main conclusions. First, as any error introduced by recombination map divergence would add noise to our analysis, we would be less likely to detect relationships, not more likely to find spurious ones. Second, the consistency of our results across phylogenetic groups, despite their varying divergence times from humans, suggests that recombination map conservation is sufficient for detecting the patterns we observe. Third, the systematic scaling with Ne that we observe cannot be explained by recombination map errors, which would affect all species similarly regardless of population size. Fourth, our comparison with the Macaca mulatta recombination map [31] demonstrates substantial conservation at the 100 kb scale in Old World monkeys.

### Linked selection leads to conservative estimates of effective population size

Our estimation of effective population size relies on nucleotide diversity from the highest recombination bins, assuming these regions best approximate neutral evolution. However, in species with large effective population sizes, even highly recombining regions may remain under the influence of linked selection, as evidenced by the absence of a diversity plateau (Fig. 2A). This creates a coupled estimation problem: both Ne and the magnitude of linked selection effects may be underestimated in these species. Specifically, if the highest recombination bins still experience selection in large-Ne species, our Ne estimates are conservative (underestimated). Simultaneously, the observed diversity-recombination slope underestimates the true impact of selection, as we cannot observe the full range from truly neutral to maximally selected sites. This limitation potentially attenuates signals in large-Ne species, making our analysis of the true effect of population size conservative.

### Simulations confirm qualitative predictions but overestimate the intensity of linked selection

Although our simulations establish qualitative expectations, direct quantitative comparison with empirical values requires caution due to the use of unrealistically high deleterious and adaptive mutation rates ($\upmu$=1e^−8^) to ensure sufficient linked selection events occur during the simulated timeframe. A back-of-the-envelope calculation illustrates the degree of inflation. In humans, each individual receives approximately 70 new mutations per generation [8], corresponding to a per-site mutation rate of ~ 1.2 × 10⁻⁸. Comparative genomic studies estimate that approximately 5% of the mammalian genome is under purifying selection [35], with estimates of ~ 1.6 new deleterious mutations arising per diploid genome per generation [36]. This yields a ratio of deleterious to total mutations of approximately 1.6/70 ≈ 2%. In our simulations, this ratio is 1 × 10⁻⁸/1 × 10⁻⁷ = 10%; roughly fivefold higher than reality. More critically, because deleterious mutations are applied uniformly across the simulated region rather than restricted to the ~ 5% of sites under selection, the effective rate of deleterious mutations per site is approximately 18-fold higher than in real genomes (1 × 10⁻⁸ vs. ~ 5.5 × 10⁻^10^). This inflated rate means that our simulations experience substantially more intense background selection than natural populations.

Assessing the realism of hitchhiking simulations is more complex. In our simulations, adaptive mutation rates are unrealistically high, resulting in many concurrent sweeps across the simulated region. This creates conditions for Hill-Robertson interference [37], where selection at multiple linked loci interferes with each other, potentially causing each individual sweep to be less impactful on diversity than in a more realistic setting where beneficial mutations arise rarely and sweep to fixation in relative isolation. This likely causes our simulations to underestimate the strength of the diversity-recombination relationship. Further, because sweeps are so frequent, even high-recombination regions cannot fully escape the effects of linked selection, as evidenced by the lack of a clear plateau at high recombination rates (Additional file 1: Fig. S8 and Fig. S12). In natural primate populations with lower beneficial mutation rates, we expect sweeps to be rarer and to produce classic hard sweeps with larger individual footprints, allowing high-recombination regions sufficient time between sweeps to recover toward neutral diversity levels, likely producing a stronger contrast between high and low recombination regions than observed in our simulations.

While our simulations show similar scaling of diversity reduction with Ne under both background selection and hitchhiking, there is reason to believe that our simulations more accurately capture the effects of background selection than hitchhiking. In our background selection simulations, diversity approaches neutral expectations at high recombination rates, suggesting that the full range of background selection impact is captured within our simulated parameter space (Additional file 1: Fig. S7 and Fig. S11). In contrast, our hitchhiking simulations fail to reach a neutral plateau even at the highest recombination rates (Additional file 1: Fig. S8 and Fig. S12), indicating that the frequent sweeps in our simulations prevent any genomic region from fully escaping the effects of linked positive selection. This saturation effect likely compresses the diversity-recombination relationship, underestimating the true contrast between high and low recombination regions that would emerge under more realistic conditions with rarer, isolated sweeps. Combined with empirical evidence that the rate of strong selective sweeps increases with population size in great apes [38], this suggests that positive selection may contribute more to the Ne-dependent patterns we observe empirically than our interference-dominated simulations would indicate. Nevertheless, the key qualitative predictions are robust across parameter ranges: linked selection produces the diversity-recombination relationship, this relationship scales with Ne, and neutral evolution cannot produce these patterns.

## Conclusion

We found a pervasive effect of linked selection across 178 primate species, causing nucleotide diversity to be reduced in all genomic regions, including those with high recombination rates. However, the relative magnitude of this effect differs dramatically among species. Their estimated effective population size explains most of these differences, with a larger population size causing more loss of diversity in low-recombining regions. Our findings complement research by Corbett-Detig, Hartl, and Sackton (2015), showing stronger linked selection in species with larger population sizes. We find that Tajima’s D is reduced in low recombination regions for all species where we had enough samples to investigate this. This suggests that linked selection is pervasive across primates. In the context of Lewontin’s paradox, our observation that nucleotide diversity varies 67-fold while effective population size varies 73-fold is easily explained by the increasing impact of linked selection with increasing population size. However, this is still a modest scale compared to the full scope of Lewontin’s paradox, where census sizes vary over many orders of magnitude.

## Methods and materials

### Variant files and lift-over

Data from 178 species, where data were readily available, and around 500 individuals were obtained from the primate diversity consortium [2], in the form of VCF files containing multiple species mapped to their closest reference genome [2]. An overview of samples and species used in the analysis is in Additional file 2: Table S2. To utilize the human pedigree-based recombination map [28], the VCF files were lifted to the hg38 coordinate system [39]. Lift-over was performed using Crossmap [40]. Chainfiles for the lift-over were likewise obtained from the primate diversity consortium [2] and based on a multispecies alignment. The number of variants mapped to the human genome is listed in Additional file 2: Table S3, including the fraction of variants lost during the lift-over.

### Estimating nucleotide diversity

After lift-over, we filtered out the X chromosome for each species and excluded all sites that had more than two segregating alleles or sites that were fixed for the reference allele. Afterwards, the average nucleotide diversity (π) was calculated for 100 kb windows using scikit-allel [41]. A similar approach was used to calculate Tajima’s D for species with more than five samples.

### Callability and filtering

For each 100 kb window, we applied a callability mask to each VCF based on multiple criteria: mode of coverage, missing genotypes, minimum threshold for alternate allele depth in heterozygous genotypes, genotype quality scores, and the fraction of the window that could be lifted over to hg38. Our filtering procedure removed regions with coverage less than half or more than twice the mode, sites with missing genotypes, sites with allelic depth below 3 for heterozygote calls, and sites with genotype quality scores below 30. Using BEDtools [42], we constructed a BED file containing the 100 kb intervals and the fraction of ‘callable’ sites (those both liftable and passing our filters). To ensure reliability while maintaining adequate representation across species, we excluded windows with less than 50% callability from further analysis. This approach retained 54–96% of windows across species, with retention generally decreasing with phylogenetic distance from humans (Additional file 2: Table S3).

### Recombination rate estimation

The recombination rate per window was estimated using the sex-averaged, pedigree-based human recombination map [28]. For each window, we inferred the relative position of the start and end of the window in centiMorgans, using the bordering positions from the recombination map, and then calculated the recombination rate in centiMorgans per megabase.

### Statistical modelling

Statistical modelling of population size, relative change, and impact of linked selection is described in detail in Additional file 1: Text. All models were fitted using PyMC3, a Bayesian framework [43]. Model comparisons were conducted using ArViz [44]. Generation times for the species were collected from Kuderna et al. [2] and COMBINE dataset [45].

### Linked selection simulations

To simulate the effects of linked selection: we used SLiM [32] and simulated the human chromosome 12 together with the recombination map [33] as implemented in the stdpopsim resource catalogue [34]. We used the distribution of fitness effects (DFE) from Kim et al. [46] to simulate the effects of background selection (a gamma with mean = −0.013, shape = 0.186) and a constant s = 0.1 to simulate strong hitchhiking. Both simulations were assuming a mutation rate of 1e-7, a runtime of 10,000 generations and for 5 different population sizes. In an effort to reach equilibrium we multiplied a constant c to the recombination rate. All simulations were run for c = 1,10,30,100. Neutral mutations were added using recapitation [47–50]. As a control we also simulated a neutral scenario.

## Supplementary Information

Additional file 1: Supplementary notes and figures.

Additional file 2: Supplementary tables.

## Data Availability

Data were obtained from the primate diversity project [2], with sequences accessed from the European Nucleotide Archive under the accession number PRJEB49549 [51]. All code and workflows are available from under a MIT License at GitHub (https://github.com/Bjarke-M/DiversitynRecombinationv2) or from Zenodo with the https://doi.org/10.5281/zenodo.19439959 [52].
