# Supplementary material for: Ubiquitous impact of natural selection on nucleotide diversity in 178 species of primates
Source: Genome Biol. 2026 May 1;27:203. doi: 10.1186/s13059-026-04093-z (PMC13281644; doi:10.1186/s13059-026-04093-z)
Supplement: Supplementary file 1 — Additional file 1: Supplementary notes and figures. [file 13059_2026_4093_MOESM1_ESM.pdf]

Supplementary

Notes

Population size modelling

The modelling of Population size is greatly inspired by Bergman et al. (2023).
Estimates of population size are difficult to attain from literature; here we chose to model population size as the effective population size of the species, a measure thought to be more relevant for evolution, and under some circumstances thought to be proportional with the census size. To model effective population sizes we need information on the mutation rate and neutral diversity. Mutation rates are generally not available for most primates. Fortunately, we can utilize the known relationship between generation time and mutation rates. To fit the model, we used data from Bergeron et al (2023), which measured average generation times and mutation rates from multiple mammals. Specifically, we fit our model only using the primate data. Our best-fitting model is a slightly modified version of the one used in Bergman et al (2023) and is as follows:

$$M_i \sim N(\mu_i, \sigma)$$
$$\mu_i = \alpha + \beta G_i$$
$$\beta \sim N(0,0.1)$$
$$\alpha \sim HalfNormal(0.1)$$
$$\sigma \sim exp(0.001)$$

Here M_i is the observed mutation rate per generation for a specific primate species i and G_i the species-specific log scaled generation time. The Mutation rate (M) and the prior on the slope (beta) are assumed to be normally distributed, while the prior on the intercept (alpha) is expected to be half normal, as mutation rates never take negative values. We have compared this model with alternative models, one with a natural scale generation time and one with a log scaled G but a normal alpha prior. To evaluate the model choice we compare the expected log predictive density for leave-one-out cross-validation (elpd loo) for all models. The models with log-scaled generation time had better predictive accuracy than the model with a natural-scale generation time, but two log-scaled models performed very similarly, with the halfnormal-alpha model having an elpd loo score of 259.61 +- 2.514 and the normal-alpha prior scoring 259.15 +- 2.109. As the half normal model elpd loo score is *slightly* higher, we use the posterior distribution from this model to estimate the mutation rates for each species, using generation times estimates obtained from literature (Kuderna et al. 2023), resulting in a distribution of mutation rates for each species. To estimate effective population size, we estimated the neutral diversity for each species, as the average nucleotide diversity in the 5% highest recombining regions, as these regions are least expected to be affected by selection, and used the relationship between diversity and mutation rate to calculate the effective population size.

$$\theta/4\mu = Ne$$

This resulted in a distribution of effective population sizes for each species, where we use the medians of these distributions when estimating the effect of population size on the impact of linked selection.

Modelling all recombination rates and diversity across the entire genome

Here we modelled the diversity (π) in each species as a function of the recombination rate (ρ) and the species' effective population size (Ne). Before inference, effective population size and recombination rate were log-scaled and diversity was standardized. The model allows for a different intercept and slope of the diversity ~ recombination rate relationship across species, but incorporates the effect of population size:

$$\pi_i \sim N(\mu_i\,,\sigma)$$
$$\mu_i \;=\; \alpha_j + \beta_j\rho$$
$$\beta_j \sim N(\gamma_j\,,\zeta)$$
$$\alpha_j \sim N(\omega_j\,,\eta)$$
$$\gamma_j \,\sim H_0 \,+\, H_1 Ne_j$$
$$\omega_j \sim G_0 \,+\, G_1 Ne_j$$
$$H_0\,, H_1\,, G_0\;\; G_1 \sim N(0{,}1)$$
$$\eta, \zeta \sim HalfNormal(1)$$
$$\sigma \sim HalfNormal(0.01)$$

Where π_i is the average nucleotide diversity at a specific recombination rate ρ_i. π_i is normally distributed with a mean μ_i and standard deviation σ. The mean μ_i is modelled as a function of the recombination rate with a slope (β_j) and intercept (α_j) that can change between species (j). Species-species specific slopes and intercepts were both modelled as response variables of a nested linear regression with effective population size as explanatory variable (Ne_j). Species-specific slopes and intercept are assumed normally distributed with mean γ_j and ω_j and standard deviation ζ and η, respectively. The hyperpriors H and G, where given normal priors. All standard deviations were given half-normal priors.

Analysing conservation of the recombination landscape

To compare the conservation of the recombination landscapes outside the apes, we lifted over the Macaca Mulatta recombination map from (Versoza et al. 2024). Then we divided each chromosome into 100kb windows, for which the recombination rate per base pair was interpolated as the weighted mean of the window. This can be imagined as if the 100kb window had 43kb with a recombination rate of 1e-8 and 57kb with a recombination rate of 1e-10, the mean of the window would be calculated as ((1e-8*43kb)+(1e-10*57kb))/100kb. Hereafter, Spearman's rank correlation was calculated both as a genome-wide measure and per individual chromosome. The correlations are in the Supplementary table S4 and the code for comparing these recombination maps is in the script 'recombination_map/recombination_conservation_analysis.ipynb' on the associated github (see Data availability).

Figures

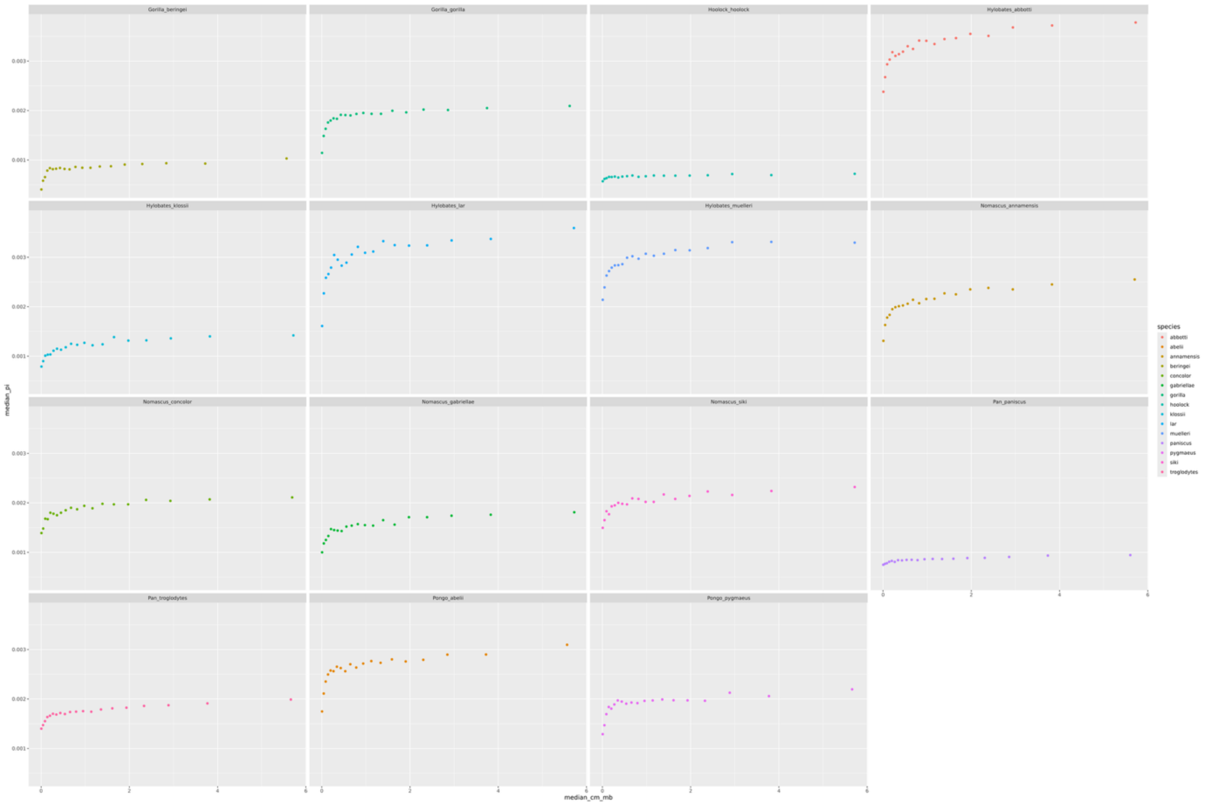

Figure S1. Recombination rate diversity relationship for each of the ape species.

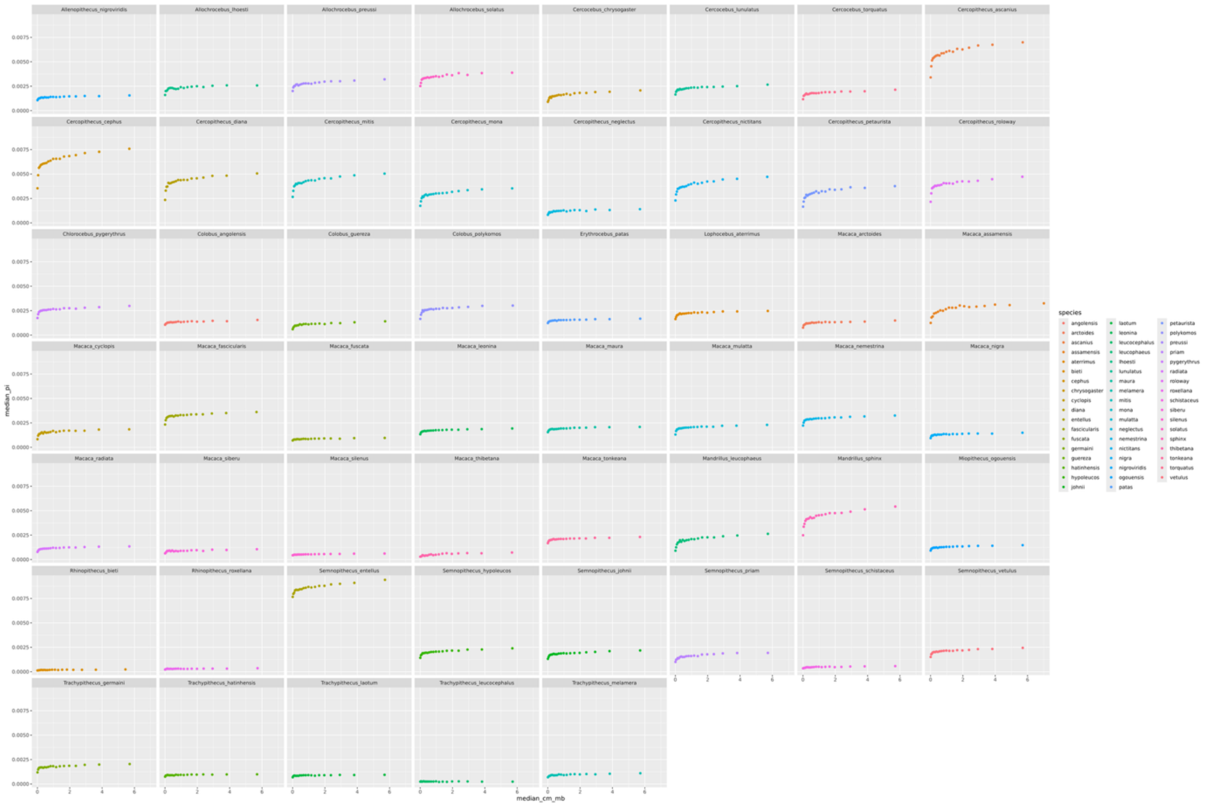

Figure S2. Recombination rate diversity relationship for each of the new world monkey species.

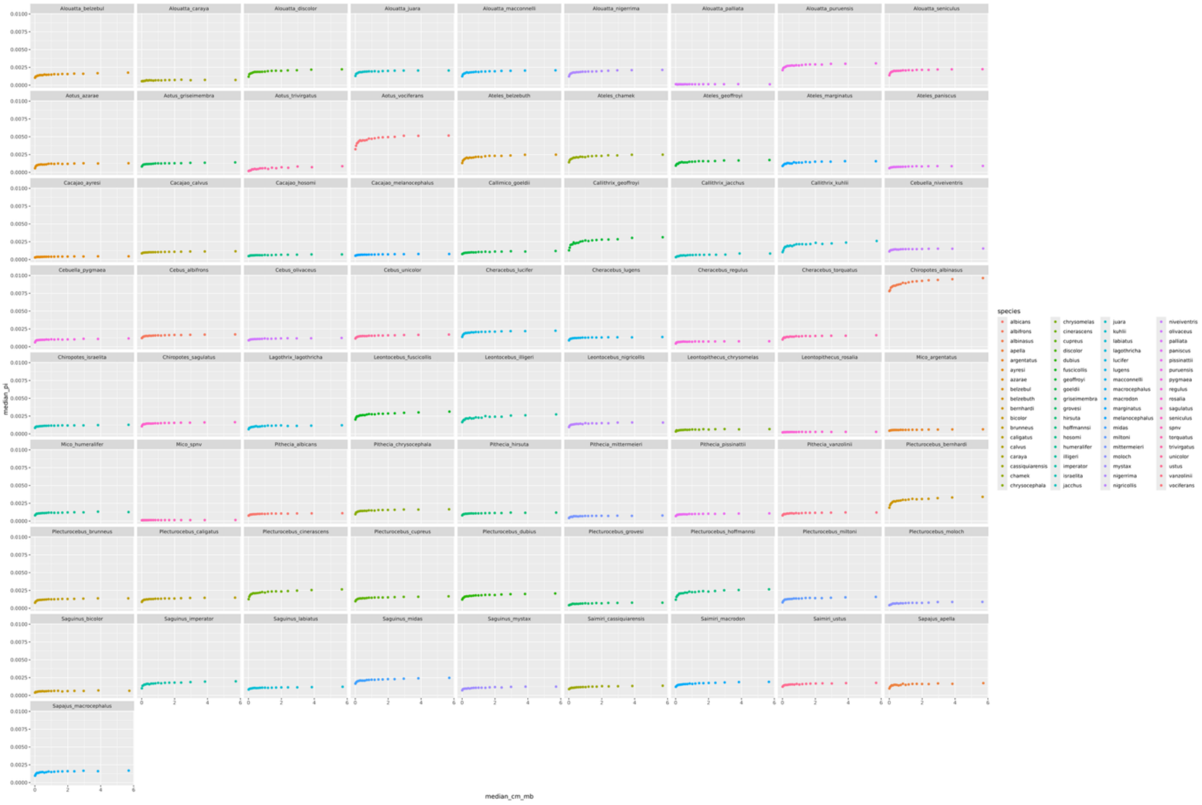

Figure S3. Recombination rate diversity relationship for each of the old world monkey species.

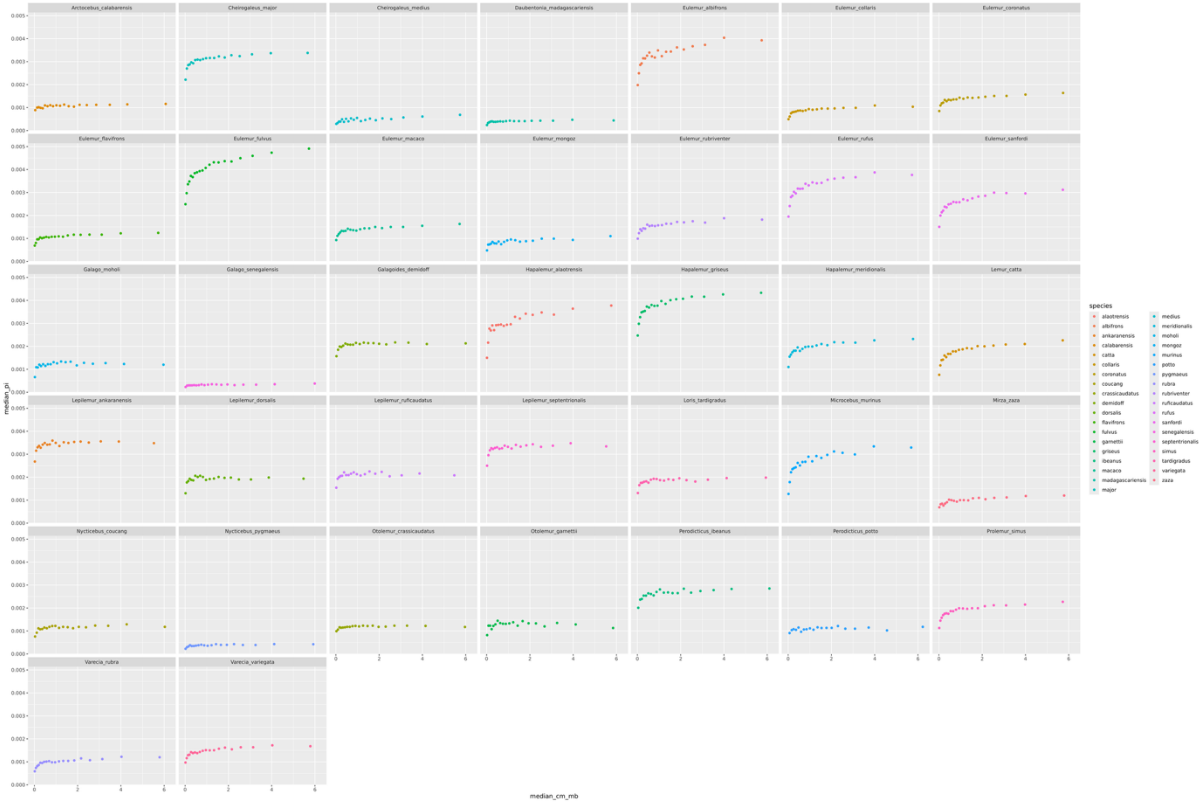

Figure S4. Recombination rate diversity relationship for each of the Prosimian species.

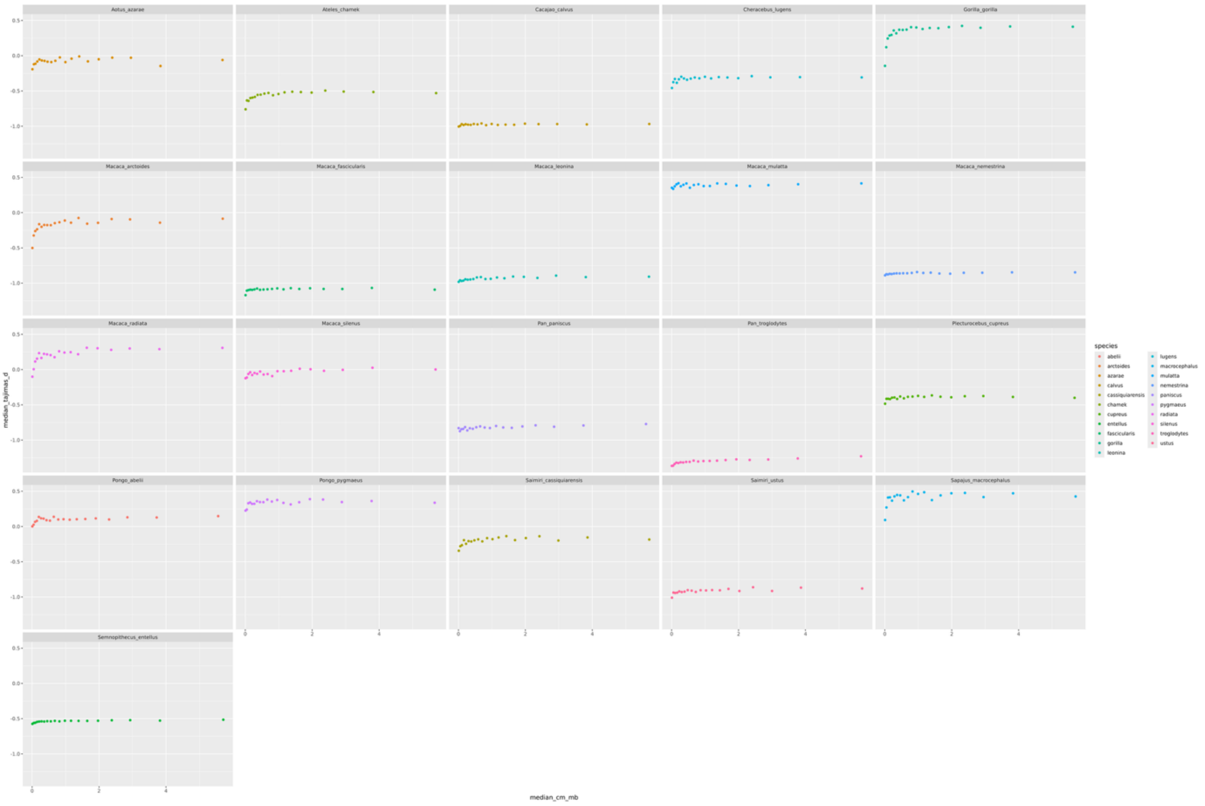

Figure S5. Recombination rate Tajima’s D relationship for all species with more than 5 samples.

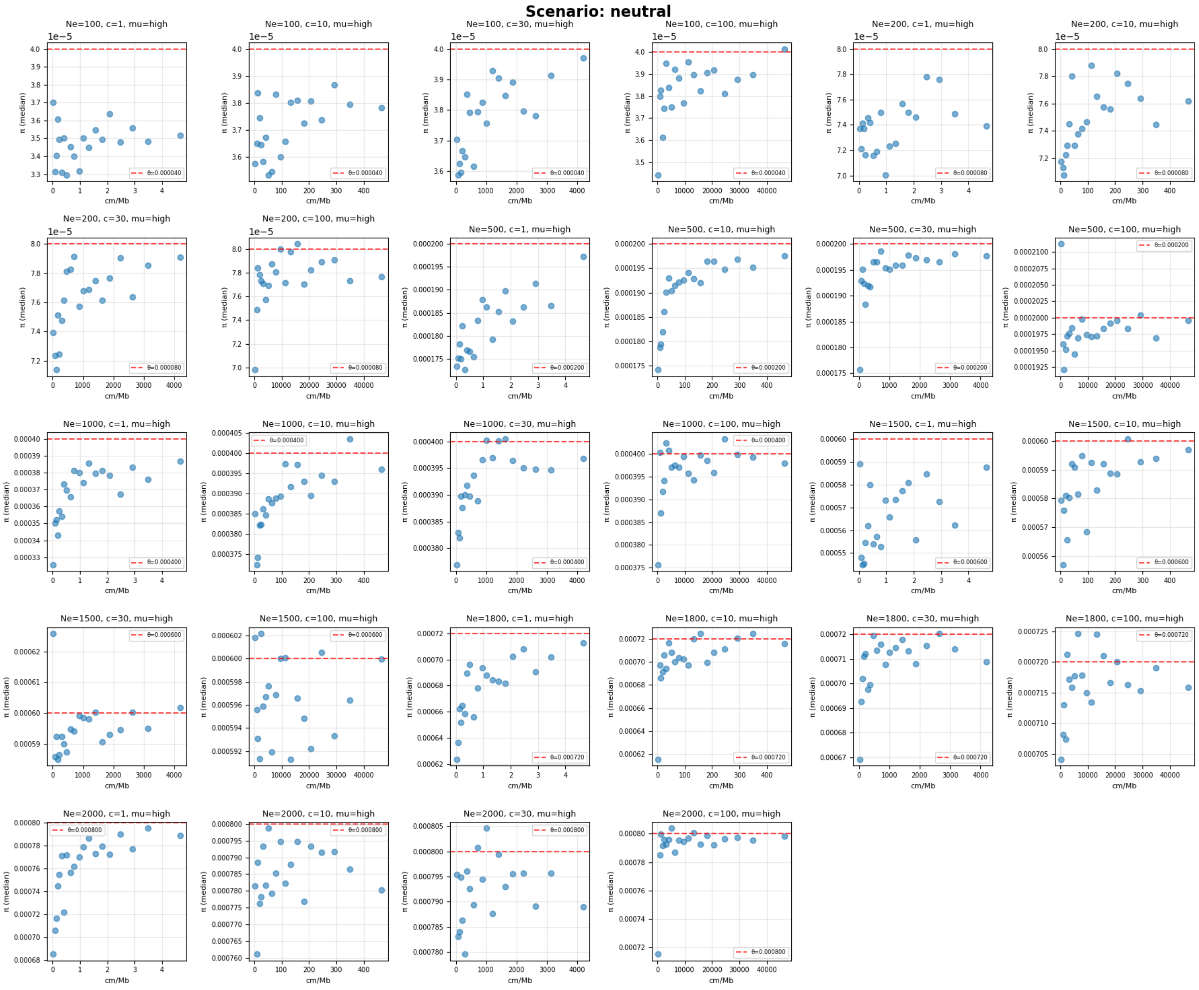

Figure S6. Diversity ~ Recombination rate in neutral simulations at different effective population sizes and different amounts of recombination.

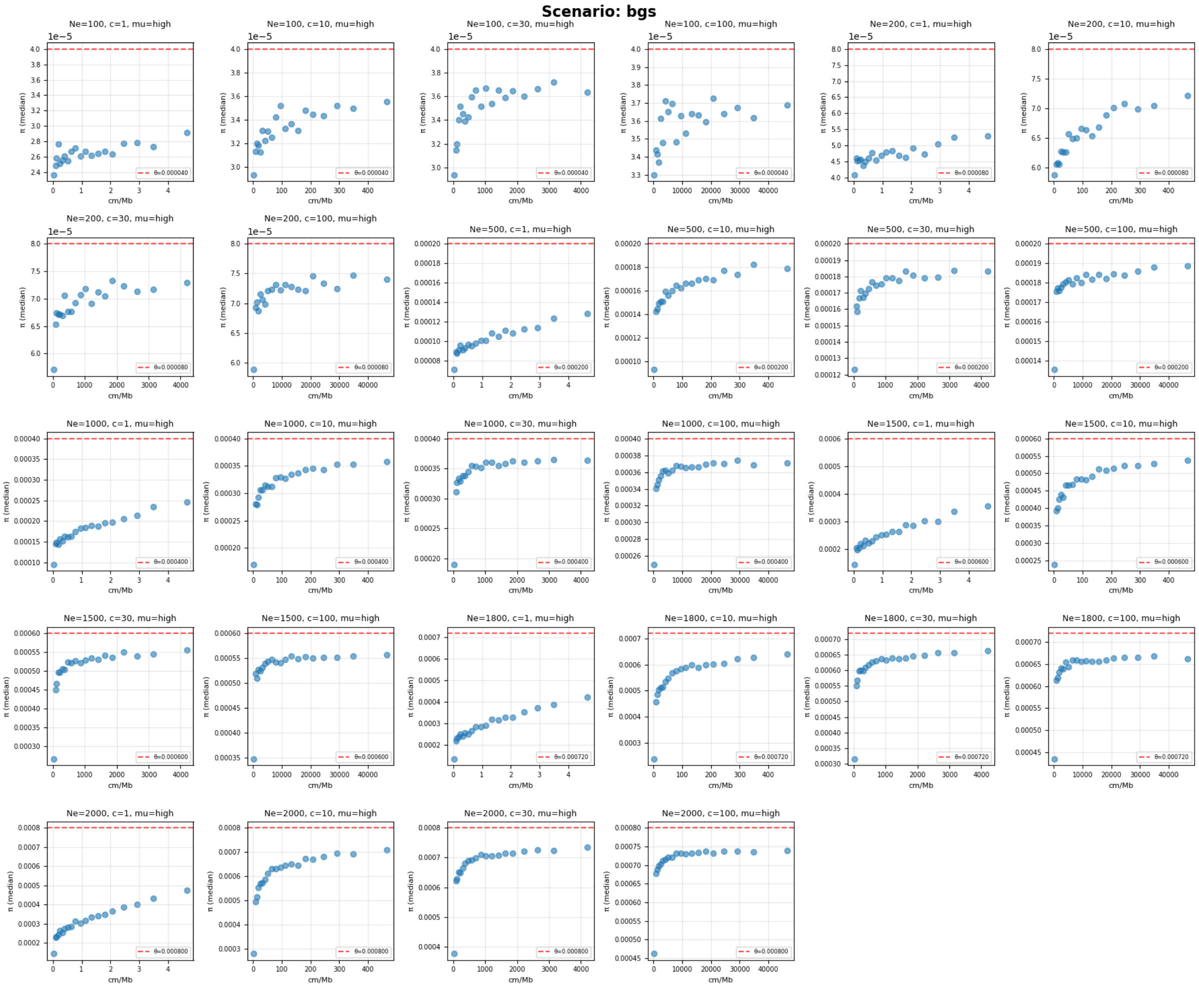

Figure S7. Diversity ~ Recombination rate in BGS simulations at different effective population sizes and different amounts of recombination.

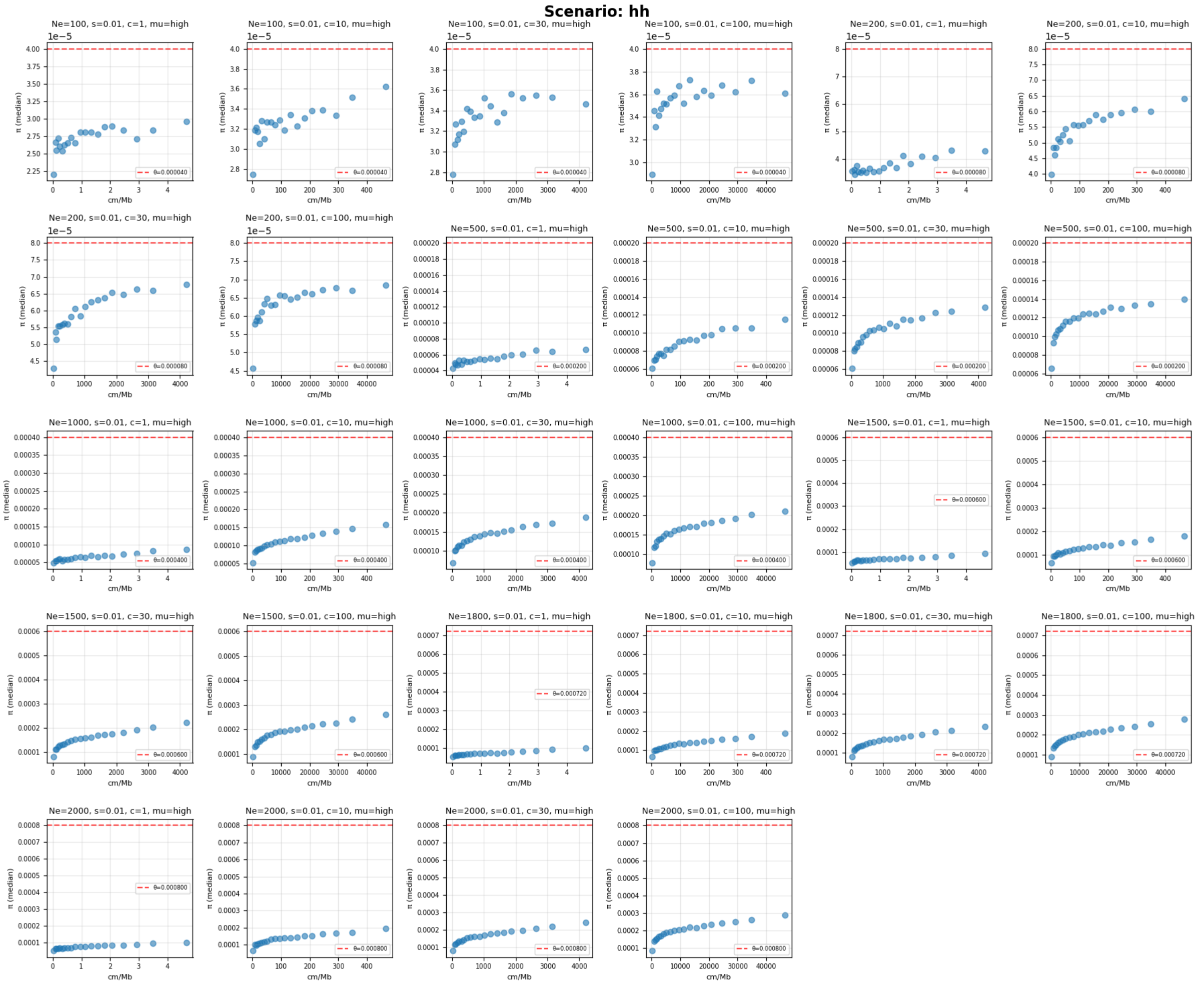

Figure S8. Diversity ~ Recombination rate in hitchhiking simulations at different effective population sizes and different amounts of recombination.

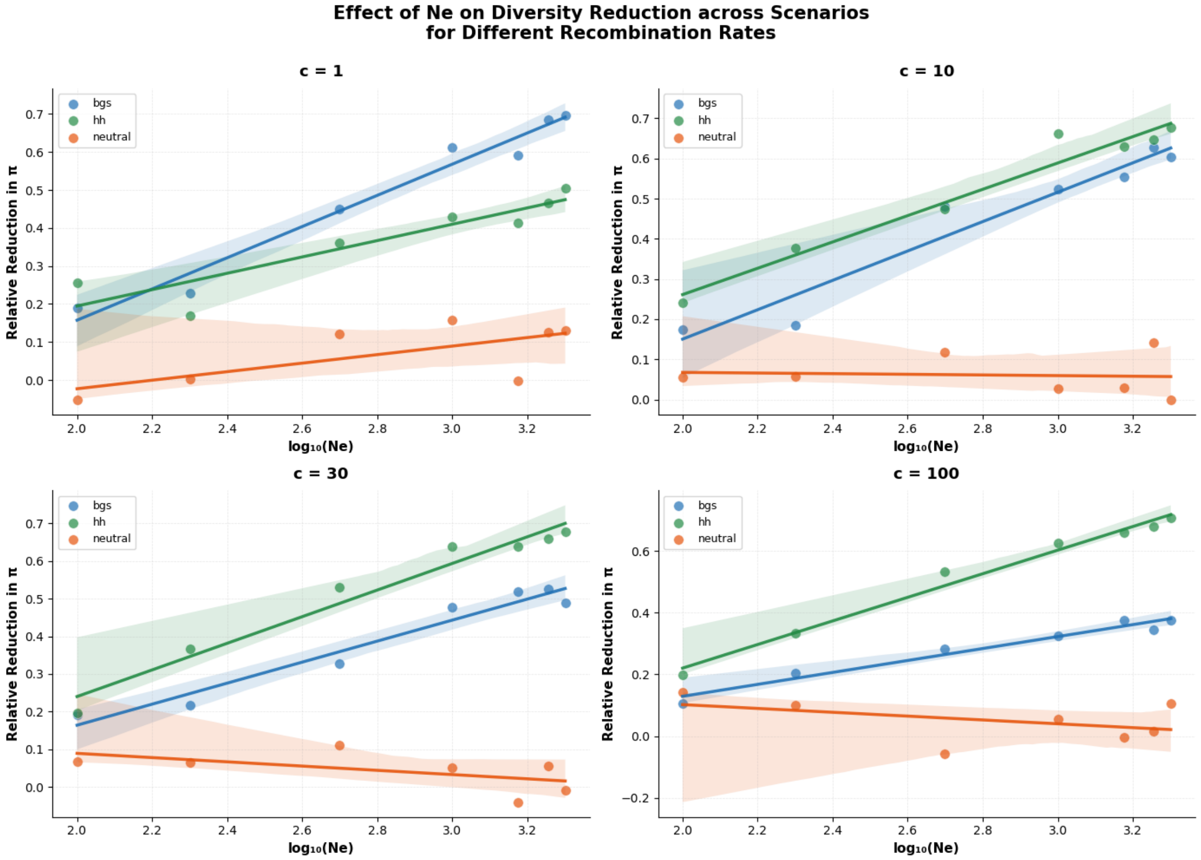

Figure S9. Relative reduction in nucleotide diversity from simulations under different selection regimes at different effective population sizes and different amounts of recombination (c=scaling factor).

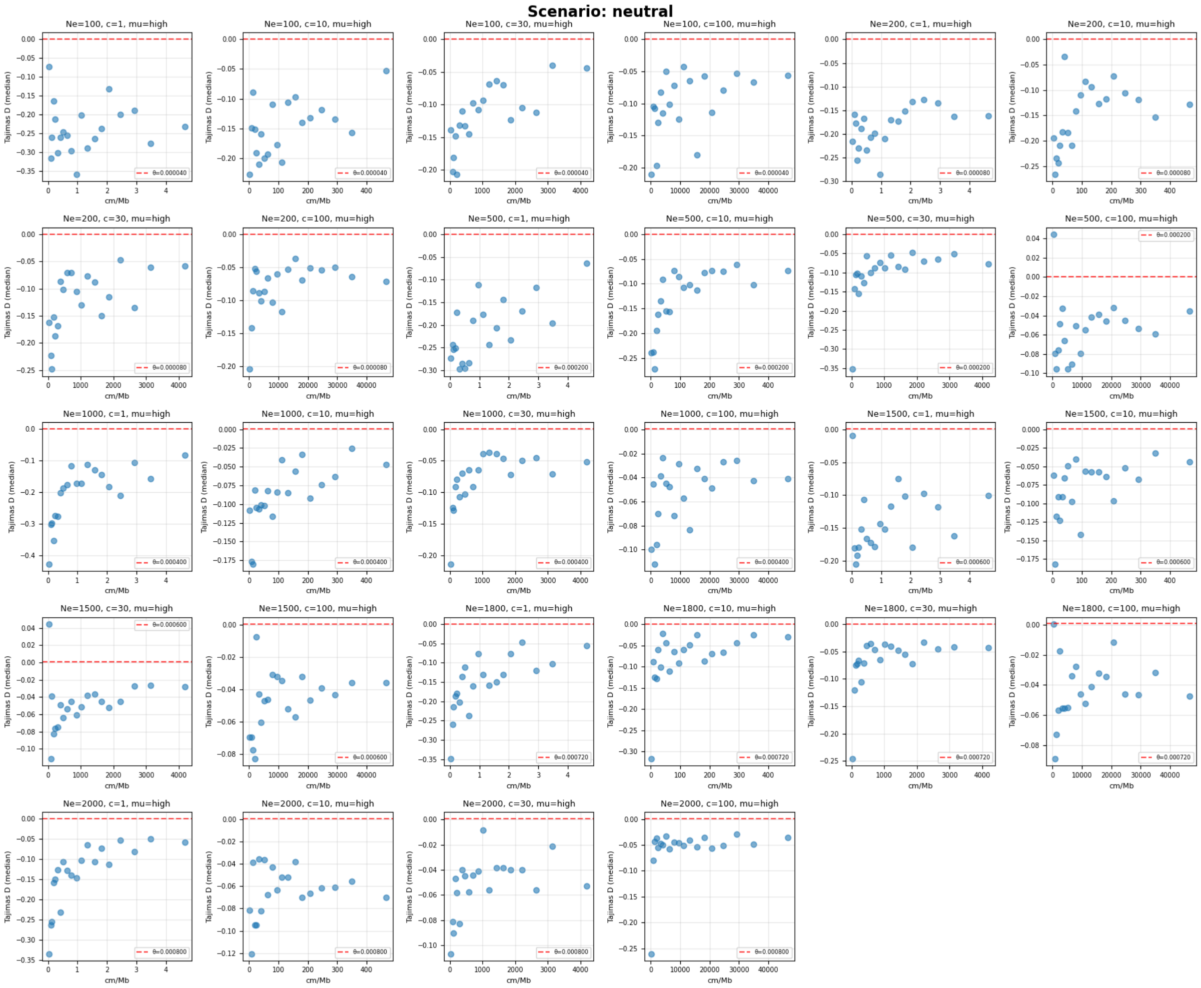

Figure S10. Tajima's D ~ Recombination rate in neutral simulations at different effective population sizes and different amounts of recombination.

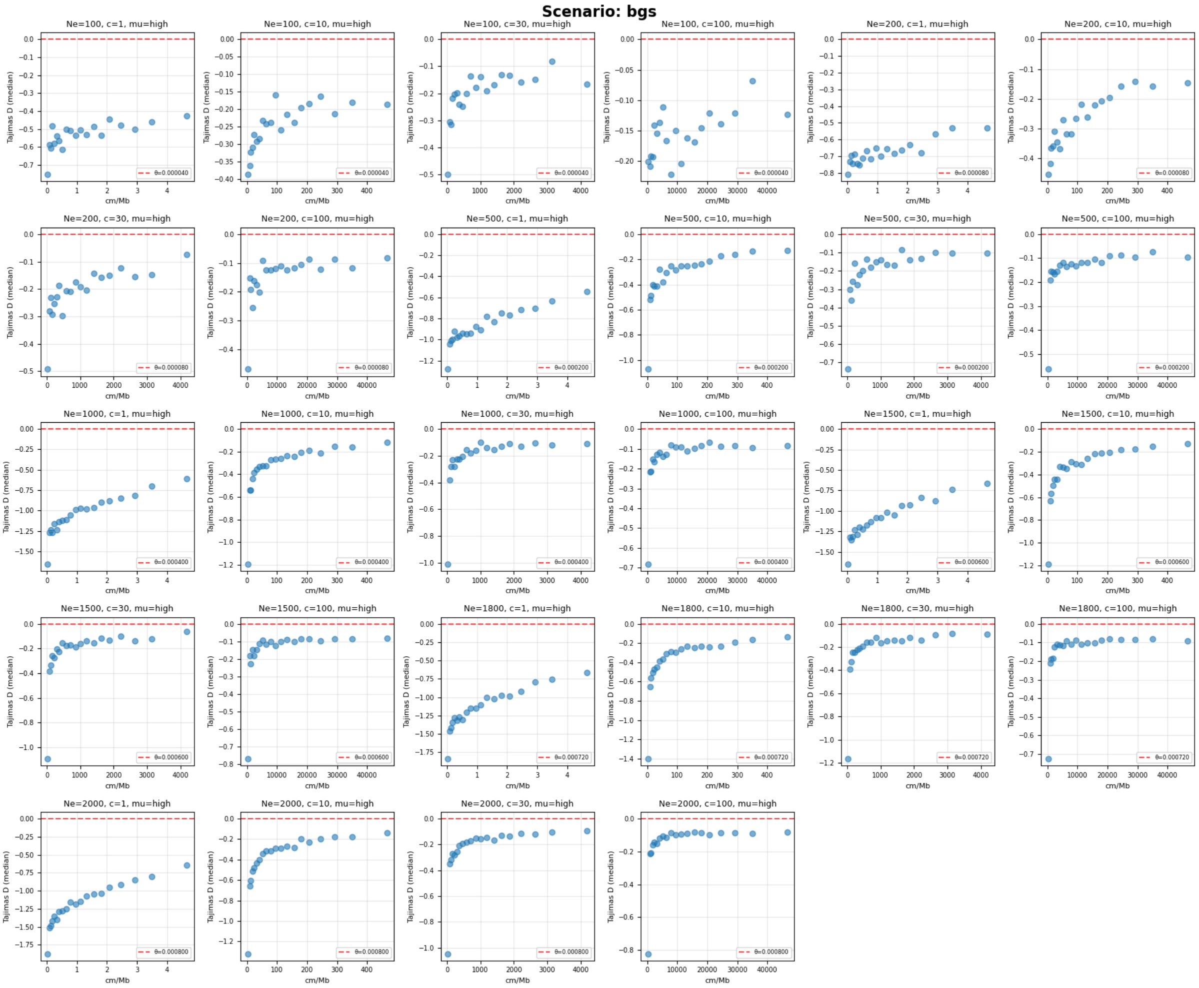

Figure S11 Tajima's D ~ Recombination rate in BGS simulations at different effective population sizes and different amounts of recombination.

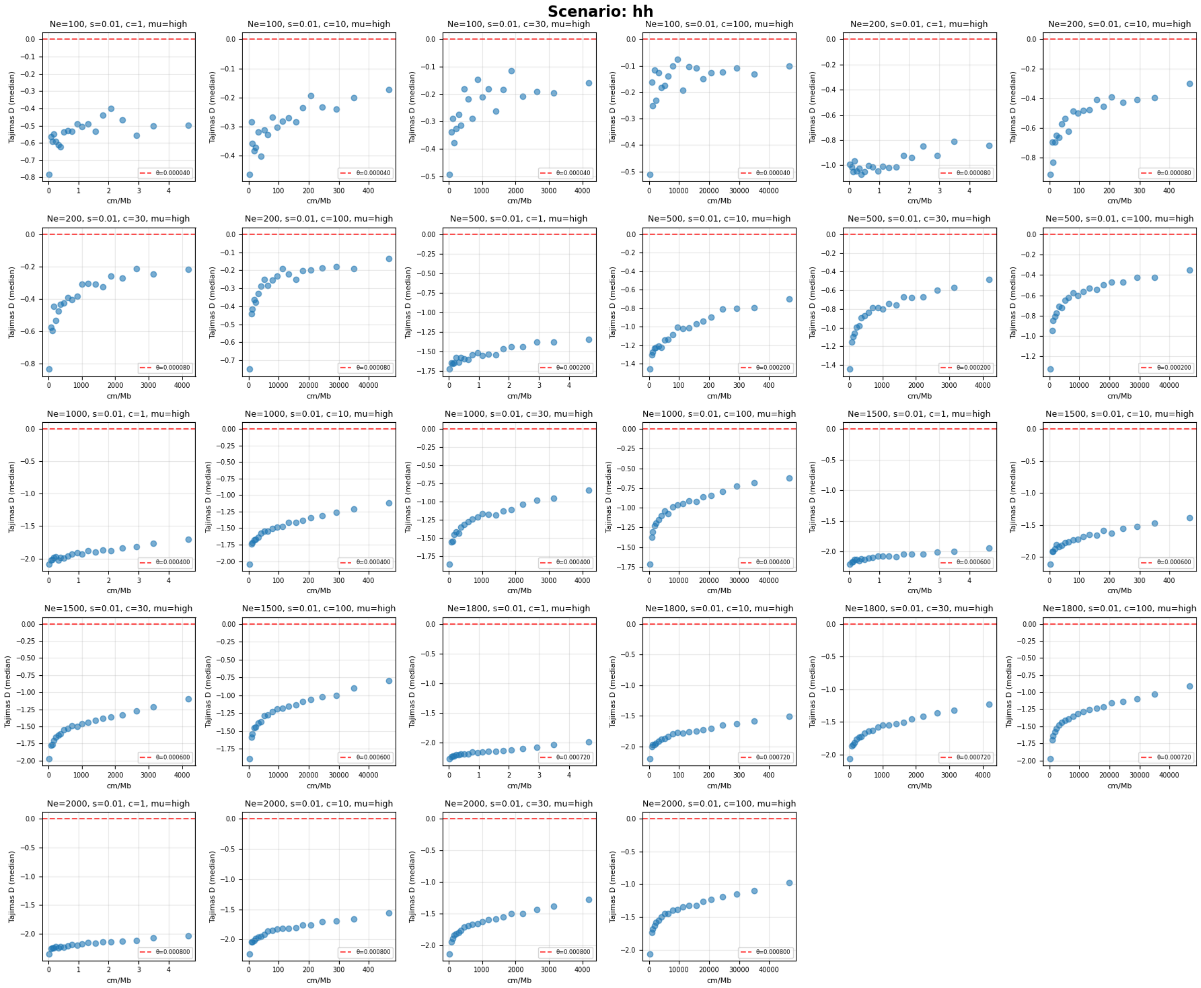

Figure S12. Tajima's D ~ Recombination rate in hitchhiking simulations at different effective population sizes and different amounts of recombination.

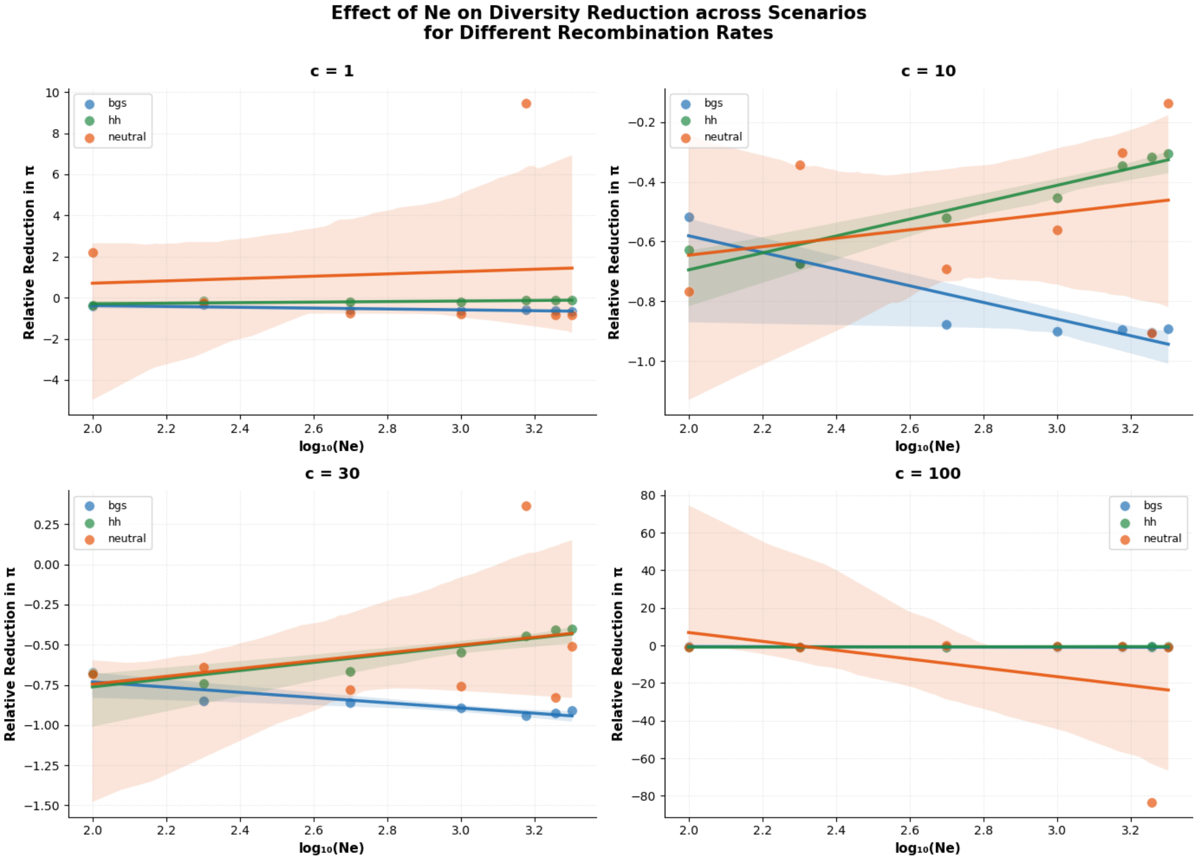

Figure S13. Relative reduction in Tajima's D from simulations under different selection regimes at different effective population sizes and different amounts of recombination (c=scaling factor).
